# Noise-aware dynamic convolution for improved generalizability of retinal disease diagnosis using optical coherence tomography images

**DOI:** 10.1117/1.JBO.31.8.086006

**Published:** 2026-08-12

**Authors:** Deeksha Chutani, Phaneendra K. Yalavarthy

**Affiliations:** a Indian Institute of Science, Department of Computational and Data Sciences, Bangalore, Karnataka, India; b Indian Institute of Science, TANUH-AI Centre of Excellence in Healthcare, Bangalore, Karnataka, India

**Keywords:** optical coherence tomography, deep learning, generalizability, dynamic convolution, retinal disease classification, conformal prediction

## Abstract

**Significance:**

Optical coherence tomography (OCT) is widely used for the diagnosis of retinal diseases. However, deep learning models trained on a single dataset often degrade when deployed across scanners and clinical sites due to device-dependent speckle variability and acquisition differences, limiting their reliability in real-world screening.

**Aim:**

We aim to develop a lightweight deep learning framework that leverages speckle characteristics in OCT images to improve cross-scanner generalizability for retinal disease classification while preserving real-time inference efficiency.

**Approach:**

We propose NA-DyCNN, a noise-aware dynamic convolutional neural network that minimizes the expected classification risk over multiple stochastic realizations of multiplicative speckle perturbations and regularizes the dynamic routing mechanism to produce scanner-invariant kernel mixtures. The framework was evaluated using over 105,000 B-scans from three heterogeneous OCT cohorts under strict zero-shot cross-dataset transfer.

**Results:**

NA-DyCNN consistently outperformed lightweight baselines across four zero-shot cross-dataset transfer scenarios, achieving up to 92.87% accuracy, a weighted $F_{2}$ score of 92.89%, and Cohen’s κ of up to 0.896, demonstrating improved robustness and generalization under cross-dataset shifts. The model maintained high efficiency with only 0.4 M parameters and an inference latency of 0.53 ms per B-scan on an NVIDIA GB10 GPU.

**Conclusions:**

Modeling postacquisition speckle variability during training improves the generalizability of OCT classifiers without increasing the inference cost, thereby enabling the more reliable deployment of artificial intelligence (AI)-assisted retinal screening across heterogeneous imaging systems.

## 1 Introduction

Optical coherence tomography (OCT) is an optical imaging modality based on low-coherence interferometry that enables noninvasive micrometer-resolution visualization of retinal microstructures, supporting the diagnosis and monitoring of diseases, such as age-related macular degeneration and diabetic macular edema.^1^^,^^2^ A fundamental characteristic of OCT imaging is the presence of speckle patterns arising from the coherent interference of backscattered light from subresolution tissue scatterers. These granular intensity fluctuations occur due to constructive and destructive interference of scattered optical waves and are therefore intrinsic to all coherent imaging systems. Importantly, OCT speckle exhibits a dual role: while it reduces visual image quality and contrast, its statistical properties encode information about tissue microstructure and optical scattering characteristics.^3^ Consequently, speckle patterns are increasingly recognized not merely as noise but as signal-carrying features that may contain diagnostically relevant information.

The increasing global burden of retinal disease has motivated the widespread adoption of deep learning for automated OCT analysis, with convolutional neural networks (CNNs),^4^ transfer learning approaches,^5^ and Vision Transformer-based models^6^ achieving strong performance on curated OCT benchmarks.^7^

Despite these advances, most reported results have been obtained under single-dataset conditions and fail to reliably translate to new scanners and clinical sites. In real-world deployments, OCT systems differ in terms of optical design, coherence length, detector sensitivity, beam scanning strategies, and reconstruction pipelines. These factors influence the statistical characteristics of speckle patterns and image contrast, leading to systematic variations in OCT appearance across devices. Consequently, models trained on a single acquisition regime frequently overfit scanner-specific texture cues and suffer substantial performance degradation under cross-site or cross-scanner transfer.^8^^,^^9^

A defining yet underutilized contributor to this domain shift is the scanner-dependent variability of the speckle patterns present in clinically archived OCT images. Speckle formation depends on factors, such as illumination coherence, optical beam properties, detector aperture, and tissue microstructure.^3^ Because these parameters vary across imaging systems, speckle statistics differ between scanners, even for anatomically similar retinas.

Historically, most OCT image processing research has treated speckle primarily as a source of signal degradation, motivating extensive work on denoising and speckle suppression through filtering, averaging, or wavelet-based methods^10^^–^^12^. However, suppressing speckle may also remove the acquisition-dependent information that characterizes the imaging system. Paluru et al.^13^ proposed a self-distillation framework to improve robustness; nevertheless, such methods remain agnostic to the physical origins of speckle formation and do not explicitly model scanner-dependent noise processes.

By contrast, the practical deployment of OCT classifiers requires models that are both computationally efficient and clinically reliable. Lightweight architectures, such as MobileNet variants,^14^ reduce inference cost but often exhibit fragile generalization under dataset shift, whereas high-capacity networks, such as ResNet^15^ are incompatible with point-of-care constraints.^16^ Moreover, clinical translation demands more than raw accuracy; calibrated uncertainty estimates and safe failure mechanisms are essential for trustworthy screening.

Dynamic convolution provides a promising compromise between expressivity and efficiency by adapting convolutional kernels to individual inputs.^17^ However, its potential to explicitly account for scanner-induced speckle variability in OCT imaging has not been investigated in prior work.

In this study, we introduce a noise-aware dynamic convolutional neural network (NA-DyCNN) for robust retinal OCT classification under scanner-induced shifts. The key contributions of this work are summarized as follows:

• **Noise-aware dynamic convolution framework.** We propose NA-DyCNN, a dynamic convolutional architecture that integrates a speckle-variability-aware training objective. By optimizing the expected classification risk over multiple stochastic realizations of post-acquisition speckle perturbations, the model learns routing functions that are robust to scanner-dependent noise characteristics while preserving inference-time efficiency.
• **Cross-dataset robustness through noise-aware training.** Unlike conventional data augmentation approaches, the proposed objective explicitly regularizes the dynamic routing mechanism by enforcing consistent kernel mixtures across multiple noise realizations of the same OCT B-scan, reducing reliance on scanner-specific texture cues.
• **Large-scale cross-dataset evaluation under strict zero-shot transfer.** We conduct a comprehensive evaluation across more than 105,000 OCT B-scans from three heterogeneous clinical cohorts (Table 1), demonstrating improved generalization across cross-scanner and cross-site domain shifts compared with static CNNs, dynamic CNNs, and lightweight mobile architectures.
• **Clinical reliability and safety analysis.** Beyond diagnostic accuracy, we evaluate calibration quality, Cohen’s κ, recall-weighted $F_{2}$ score, and uncertainty attribution using the error–uncertainty (Err.Unc) metric. We further assessed deployment safety using Mondrian conformal prediction, providing finite-sample coverage guarantees under dataset shift.

## 2 Methods and Materials

### 2.1 Datasets

We evaluated the proposed framework across three large, distinct retinal OCT datasets acquired using heterogeneous scanner hardware and clinical acquisition protocols. To enable robust cross-dataset evaluation, we restrict our analysis to four clinically significant classes: choroidal neovascularization (CNV), diabetic macular edema (DME), Drusen, and normal. A detailed summary of the dataset characteristics is provided in Table 1.

**Table 1:** OCT dataset characteristics and usage protocols after quality control (QC).

Dataset	Source/scanner	Images	Classes used	Resolution profile (post-QC)	Usage protocol
$D_{1}$ – **UCSD**	Heidelberg Spectralis SD-OCT (Multi-center)^7^	∼84 k	CNV, DME, DRUSEN, NORMAL	512 × 496 (60%), 768 × 496 (31%), 512 × 512 (9%)	In-dataset training; cross-dataset test for $D_{2}\to D_{1}$
$D_{2}$ – **OCT-C8**	Curated multivendor OCT^18^	∼10.9 k	CNV, DME, DRUSEN, NORMAL^a^	512 × 496 (∼48%), 512 × 512 (∼27%), 768 × 496 (∼25%)	In-dataset training; cross-dataset test for $D_{1}\to D_{2}$
$D_{3}$ – **NEH**	Heidelberg Spectralis SD-OCT (Noor Eye Hospital, Tehran, Iran)^19^	∼10.9 k	CNV, DRUSEN, NORMAL	Uniform 768 × 496 (100%)	External test only ($D_{1}\to D_{3},D_{2}\to D_{3}$)

a Four classes selected from original eight to ensure cross-dataset consistency.

$D_{1}$ (UCSD)^7^ was split at the patient level^20^ into 70/15/15 train/validation/test subsets after filtering rare or highly anisotropic resolutions, retaining only images of size 512×496, 768×496, and 512×512. This dataset was curated from multiple clinical centers and labeled through a multitier grading pipeline involving trained graders, ophthalmologists, and senior retinal specialists.^7^

$D_{2}$ (OCT-C8)^18^ served as an independent multivendor cohort, where only four overlapping classes were retained and the original dataset splits were preserved after identical resolution filtering. This dataset aggregates retinal OCT images from multiple sources and therefore reflects heterogeneous acquisition environments and scanner configurations.^18^

In $D_{3}$ (NEH),^19^ all images were resolution-uniform (768×496), and only B-scans whose frame-level labels matched clinician-assigned volume labels were retained to construct a patient-balanced evaluation cohort across CNV, Drusen, and normal classes. The NEH dataset contains more than 16,000 OCT B-scans acquired at Noor Eye Hospital (Tehran, Iran) and includes frame-level labels assigned by a retinal specialist.^19^ The publicly available NEH dataset does not include DME annotations; therefore, all evaluations involving $D_{3}$ were performed in a three-class setting using only overlapping diagnostic categories.

Two independent training configurations were evaluated to assess cross-dataset generalization. In the first configuration, models were trained on $D_{1}$ (UCSD) and evaluated on both $D_{2}$ (OCT-C8) and $D_{3}$ (NEH). In the second configuration, models were trained on $D_{2}$ (OCT-C8) and evaluated on $D_{1}$ (UCSD) and $D_{3}$ (NEH). Because $D_{3}$ (NEH) does not include DME annotations, models trained on $D_{3}$ would produce a three-class classifier incompatible with the four-class evaluation protocol of $D_{1}$ and $D_{2}$. Consequently, $D_{3}$ serves exclusively as a target domain in all transfer evaluations.

Although $D_{1}$ (UCSD) and $D_{3}$ (NEH) share the Heidelberg Spectralis SD-OCT platform, they differ substantially in acquisition protocols, clinical sites, patient demographics, and annotation practices. Consequently, $D_{1}\to D_{3}$ represents a cross-site shift without a scanner change, whereas all $D_{2}$ transfers involve both cross-scanner and cross-site variability.

All datasets consist of vendor-exported clinical B-scans stored in standard image formats. $D_{1}$ (UCSD) contains JPEG images, $D_{2}$ (OCT-C8) contains JPG images, and $D_{3}$ (NEH) contains JPG and TIFF images. These formats reflect typical postprocessed exports used in hospital PACS systems and telemedicine transmission.

Together, these datasets represent heterogeneous clinical cohorts acquired across different institutions, annotation pipelines, and imaging environments, providing a suitable benchmark for evaluating cross-dataset robustness of OCT classification models.

### 2.2 Image Preprocessing and Data Augmentation

Raw B-scans were standardized to 8-bit grayscale, and 16-bit inputs were linearly scaled to preserve the luminance structures. To maintain the retinal layer morphology and device-specific texture characteristics, the images were square-padded to max(W,H) before being downsampled to 256×256  pixels using Lanczos interpolation.^21^ Finally, per-image Z-normalization was applied to ensure numerical stability and contrast consistency across heterogeneous scanner hardware. During training, mild spatial (horizontal flip, rotation, affine translation, and scaling) and photometric (brightness and contrast jitter) augmentations were applied; no augmentation was used during the evaluation. All experiments were implemented in PyTorch^22^ using deterministic settings and three random seeds as a computational trade-off across multiple cross-dataset transfer settings, ablation studies, calibration analysis, and uncertainty experiments. To verify that three seeds were sufficient, we additionally evaluated the highest-variance transfer setting (dynamic CNN on $D_{1}\to D_{3}$) using five seeds. The resulting performance ($F_{2}$ score) changed only marginally from 66.78±8.9 (three seeds) to 65.37±8.7 (five seeds), indicating that the observed variability was primarily due to task difficulty rather than insufficient seed sampling. Importantly, the relative ranking of the methods remained unchanged, with NA-DyCNN continuing to outperform Dynamic CNN by a substantial margin on this challenging transfer setting (72.54±2.1 versus 65.37±8.7), suggesting that the main conclusions of the study are robust to additional seed sampling.

The OCT datasets (Table 1) used in this study contained only vendor-exported, log-compressed B-scans. Raw interferometric or linear-intensity OCT volumes required for first-principles speckle simulations are not accessible in UCSD, OCT-C8, or NEH, which constrains all learning-based methods to operate in the postacquisition image domain. Therefore, our objective was not to reproduce physical speckle formation but to model the residual speckle variability that persists after vendor-specific postprocessing and defines the cross-scanner domain shift in real clinical exports.

**Fig. 1:**
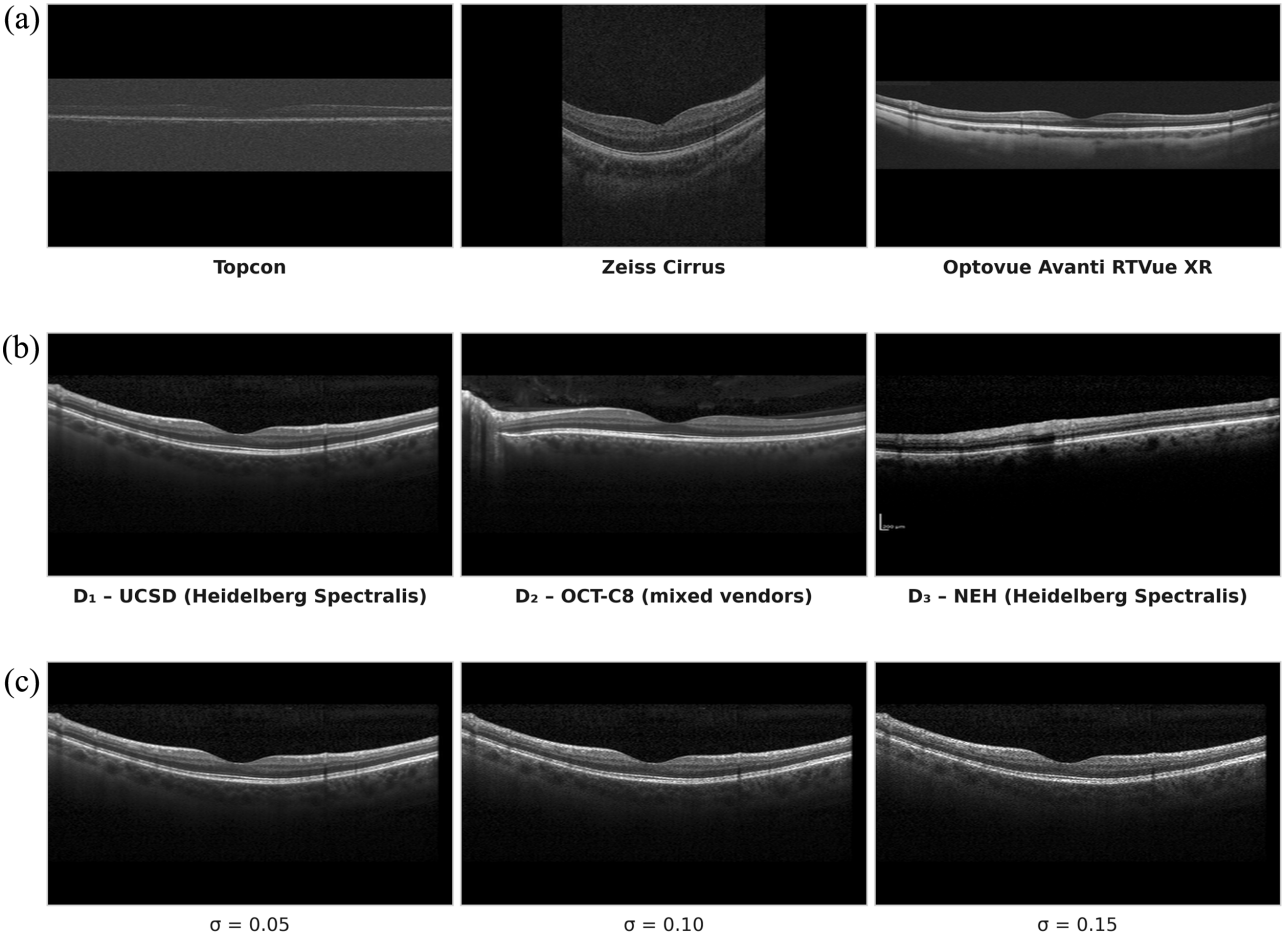
Visualization of OCT appearance variability, together with sample synthetic perturbations used during noise-aware training. (a) Representative OCT B-scans obtained from publicly available sources acquired using Topcon,^23^ Zeiss Cirrus,^24^ and Optovue Avanti RTVue XR^25^ systems, illustrating the diversity of image appearance, contrast characteristics, and retinal layer representation across different vendors. (b) Representative OCT B-scans from the three datasets used in this study: $D_{1}$ (UCSD; Heidelberg Spectralis), $D_{2}$ (OCT-C8; mixed vendors), and $D_{3}$ (NEH; Heidelberg Spectralis), illustrating scanner-dependent variations in the speckle texture and reflectivity statistics. (c) Noise-perturbed realizations of the same OCT B-scan generated using multiplicative Gaussian perturbations (σ=0.05, 0.10, and 0.15), illustrating increasing levels of synthetic speckle variability. These values are shown for visualization purpose and do not correspond exactly to the hyperparameters used during training.

To visually illustrate the cross-scanner appearance variability motivating our approach, Fig. 1 shows representative scans from different vendors in the first row followed by normal retinal OCT B-scans from the three datasets used in this study in the second row. Noticeable differences in speckle texture, background statistics, and contrast are observed across datasets due to variations in scanner hardware, acquisition parameters, and vendor-specific reconstruction pipelines. For clarity, the third row shows noise-perturbed realizations of the same UCSD normal B-scan using multiplicative perturbations with increasing variance, illustrating the type of residual speckle variability modeled during noise-aware training.

### 2.3 Baseline Methods

**Fig. 2:**
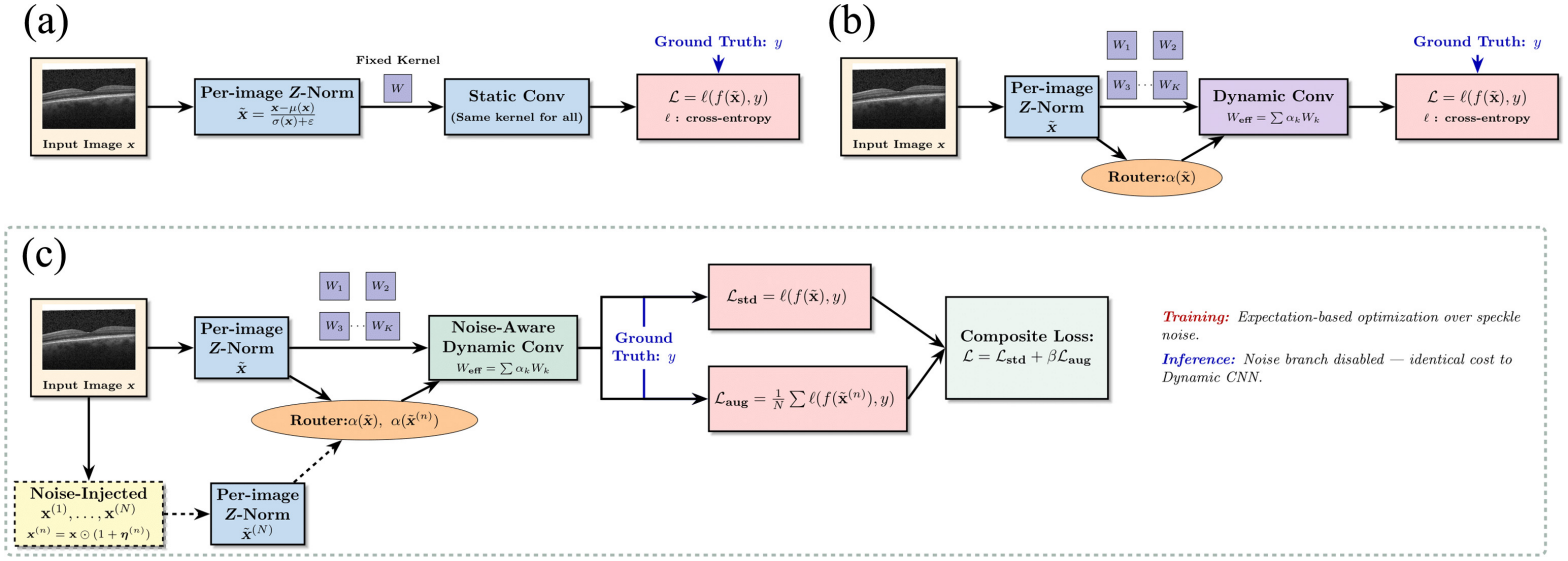
Structural comparison of convolutional architectures for OCT classification. (a) Basic CNN applies per-image z-normalization, followed by static convolution with shared kernels across all inputs. (b) Dynamic CNN employs a router network to generate image-dependent kernel mixtures for dynamic convolution. (c) Noise-aware dynamic CNN (proposed) introduces expectation-based regularization by training on multiple stochastic realizations of scanner-export speckle variability $x^{\left(n\right)}$ of each input while using the same dynamic routing mechanism for both clean and noisy samples. During inference, the noise branch is disabled, yielding a computational cost identical to that of the standard dynamic CNN. Here, ℓ(·,·) denotes the multiclass cross-entropy loss.

To validate the efficiency and robustness of our framework, we compared it with four distinct architectures. The three custom convolutional designs (basic CNN, dynamic CNN, and proposed NA-DyCNN) are illustrated in Fig. 2.

All models were trained from scratch to avoid the confounding scanner-domain bias introduced by ImageNet pretraining, which has been shown to encode nonmedical texture priors and can artificially inflate cross-domain performance.

• **Basic CNN**: A standard hierarchical baseline comprising four sequential convolutional blocks (16→32→64→128 filters) followed by global average pooling (GAP) and a two-layer MLP classifier (Fig. 2(a)). This serves as a reference for static feature extraction.
• **Dynamic CNN**: Based on Chen et al.,^17^ this model shares the identical macro-architecture and parameter count as the basic CNN but replaces static layers with dynamic convolution modules [with K=4 experts; Fig. 2(b)]. This isolates the specific contribution of the input-adaptive processing. Following prior implementations of dynamic convolution,^17^ which commonly use multiple parallel kernels per layer, we set the number of experts to four for all dynamic convolution models. This configuration provides a practical trade-off between the representational capacity and computational cost.
• **MobileNetV3-Small**: A state-of-the-art lightweight architecture^14^ utilizing depthwise-separable convolutions, included to benchmark performance against established mobile-optimized networks.
• **ResNet18 (reference only)**: A high-capacity residual network^15^ was included solely as a computationally unconstrained performance reference. It was reported only to contextualize the robustness–efficiency trade-off achieved by lightweight models.

Table 2 summarizes the parameter counts, FLOPs, empirical inference latency, and relative training cost. As illustrated in Fig. 2(c), NA-DyCNN introduces speckle-variability-aware regularization only during training; consequently, its inference-time computational cost is identical to that of the dynamic CNN. The training overhead of 4.69× relative to dynamic CNN reflects the cost of processing N=4 additional noisy forward passes per mini-batch and is incurred only during training. Because model training is performed offline once and the resulting model is deployed without the noise branch, this additional cost does not affect the real-world inference efficiency.

**Table 2:** Computational complexity and inference efficiency for 256×256 grayscale input on NVIDIA GB 10 with batch size 1. Latency measurements represent the mean ± std over 1000 forward passes. The relative training cost is normalized to dynamic CNN (1.0×), measured on the $D_{2}$ training split (batch size 64). Bold entries denote the proposed method.

Model	Params (M)	FLOPs (M)	Latency (ms)	FPS	Relative training cost	Convolution type
Basic CNN	0.11	243.8	0.25 ± 0.04	4000	0.90×	Static
Dynamic CNN	0.40	482.5	0.53 ± 0.05	1886	1.00×	Dynamic
**Proposed (NA-DyCNN)** ^a^	**0.40**	**482.5**	**0.53 ± 0.05**	**1886**	4.69×	**Dynamic**
MobileNetV3	1.52	75.2	1.41 ± 0.06	709	0.79×	Static (Depthwise-sep.)
ResNet18	11.17	2279.0	1.26 ± 0.10	793	1.73×	Static (Residual)

FLOPs are reported for B=1 and include router overhead for dynamic CNN variants.

Relative training cost is normalized to dynamic CNN (5.57±0.17  s/epoch); For NA-DyCNN, the training time is 26.13±0.29  s/epoch (N=4 realizations).

a Noise-aware training affects only the training phase; inference cost is identical to dynamic CNN.

### 2.4 Proposed Approach: Noise-Aware Dynamic Convolutional Neural Network

We propose a noise-aware dynamic convolutional neural network (NA-DyCNN) (Fig. 2) that explicitly models the postacquisition speckle variability present in clinically archived OCT images within the learning objective. Rather than optimizing on a single clean realization, the model minimizes the expected classification risk over a distribution of speckle-corrupted observations, thereby learning features and routing functions that are invariant to scanner-dependent noise statistics while preserving real-time inference efficiency.

#### 2.4.1 Motivation: postacquisition speckle variability as a source of domain shift

Retinal OCT is a coherent imaging modality^1^^,^^2^ and is inherently affected by multiplicative speckle noise arising from the interference of backscattered light from subresolution tissue structures. The statistical properties of this noise vary across scanner hardware due to differences in coherence length, detector sensitivity, and signal averaging strategies, making vendor-processed speckle variability a dominant observable source of cross-dataset shift in clinical exports. Conventional and dynamic CNNs trained on single image realizations tend to overfit scanner-specific noise signatures, degrading generalization to unseen acquisition systems.

#### 2.4.2 Realistic speckle modeling in processed clinical data

Although theoretical OCT speckle statistics are best modeled using raw 12-bit or 16-bit intensity data, standard machine learning benchmarks, including the datasets used in this study (Table 1), consist of postprocessed, eight-bit quantized images. These formats have undergone proprietary dynamic range compression (log-transformation) and bit-depth reduction, which reduces the fine-grained speckle structure. This reflects realistic clinical deployment conditions, where processed eight-bit exports are the standard for hospital storage and telemedicine transmission.

Although raw OCT acquisitions are influenced by multiple factors, including shot noise, thermal noise, readout noise, speckle effects, reconstruction artifacts, and vendor-specific processing, these components are no longer directly separable after image reconstruction and export. Therefore, the proposed multiplicative perturbation does not aim to physically simulate a specific OCT noise source. Instead, it provides a lightweight approximation of the residual acquisition-related variability observable in processed clinical OCT images, including variability arising from scanner hardware, reconstruction algorithms, image resolution, and vendor-specific preprocessing pipelines. Consequently, our noise injection strategy does not attempt to simulate raw interferometric speckle but rather models the residual speckle variability and scanner-specific noise texture remaining after clinical post-processing. Prior work has identified these residual texture differences as a persistent source of cross-device domain shifts in vendor-exported OCT images.^8^^,^^9^ By injecting multiplicative intensity variations directly into the processed domain, we effectively targeted the device-dependent signal-to-noise signatures observable in deployed screening systems. This framing reflects the practical constraint that all existing large-scale OCT benchmarks operate exclusively in the post-acquisition image domain.

#### 2.4.3 Noise-aware learning formulation

Let $x\in [0,1{]}^{H\times W}$ where H and W denote the image height and width, respectively, denote the clean OCT B-scan. Noise-corrupted realizations are generated as

$$x^{\left(n\right)}=\text{clip}(x⊙(1+\eta^{\left(n\right)}),0,1),\;\eta^{\left(n\right)}∼N(0,\sigma^{2}),$$ (1)

where noise is injected in the postexport intensity domain prior to normalization to emulate the scanner-level variability observed in clinical OCT exports.

Per-image Z-normalization is defined as

$$Z(x)=\frac{x-\mu (x)}{\sigma (x)+\varepsilon }.$$ (2)

Each dynamic convolution layer maintains a bank of K expert kernels with effective filters

$$W_{\mathrm{eff}}(\overset{˜}{x})=\overset{K}{\underset{k=1}{\sum }}\alpha_{k}(\overset{˜}{x})W_{k},\;\alpha (\overset{˜}{x})=\text{Router}(\overset{˜}{x}).$$ (3)

Here, $\tilde{x}=Z(x)$ denotes the normalized OCT B-scan. Further, μ(x) and σ(x) denote the mean and standard deviation of the pixel intensities in image x, respectively, and ε is a small constant added for numerical stability to avoid division by zero during normalization. The index n∈{1,…,N} denotes the stochastic noise realization, where N is the total number of realizations used during training. The function f(·) denotes the classifier mapping normalized OCT B-scans to class probabilities. Each dynamic convolution layer contains K expert kernels, where $W_{k}$ denotes the $k^{\mathrm{th}}$ expert kernel and $\alpha_{k}(\cdot )$ denotes its corresponding routing weight. Finally, α represents the vector of routing weights produced by the router network.

The router is implemented as a lightweight attention module consisting of global average pooling, followed by two 1×1 convolutions with a ReLU nonlinearity and a softmax activation. This design yields per-image kernel mixture weights with negligible computational overhead, ensuring that dynamic routing does not compromise the inference efficiency.

#### 2.4.4 Noise-aware objective

**Fig. 3:**
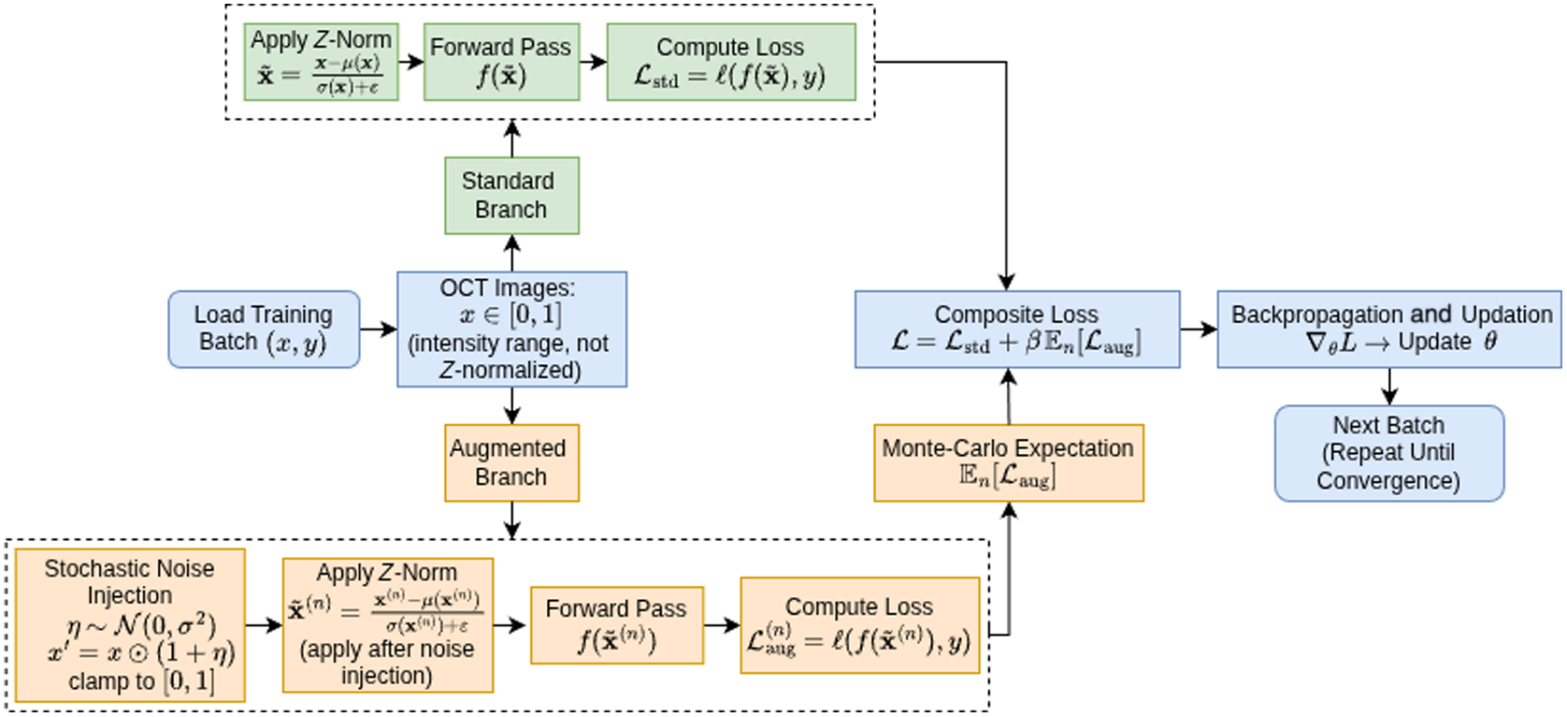
Training workflow of the proposed noise-aware dynamic CNN. Each mini-batch is processed through a standard branch producing $L_{\mathrm{std}}$ and a noise-augmented branch producing $L_{\mathrm{aug}}$, where multiplicative Gaussian noise is injected prior to per-image Z-normalization to simulate the post-acquisition scanner speckle variability. The Monte-Carlo estimate of the augmented loss is combined with the standard loss [Eq. (6)] to update shared network parameters θ, encouraging the model to learn representations that are invariant to scanner-export speckle variability across devices.

The training workflow is shown in Fig. 3. Classification is performed using the multiclass cross-entropy loss ℓ(·,·) with label smoothing to mitigate overconfidence under dataset shift. The standard loss is

$$L_{\mathrm{std}}=\ell (f(Z(x)),y),$$ (4)

whereas the noise-aware loss is approximated as

$$L_{\mathrm{aug}}=\frac{1}{N}\sum_{n=1}^{N}\ell (f(Z(x^{\left(n\right)})),y).$$ (5)

The expectation is approximated via Monte-Carlo sampling over N independent realizations of post-acquisition speckle variability per image.

The final training objective is

$$L=L_{\mathrm{std}}+\beta L_{\mathrm{aug}},$$ (6)

where β controls the strength of the noise-aware regularization.

Because multiplicative noise is injected prior to intensity clamping and per-image Z-normalization, the expected value of each noisy realization does not equal the clean input. The clamping operation introduces distributional asymmetry, and subsequent Z-normalization further differentiates the statistics of clean and noisy signals. The noise-aware objective therefore exposes the routing network to genuine input variance rather than mean-equivalent perturbations, encouraging kernel mixtures that are stable across the scanner-dependent noise neighborhood of each B-scan.

#### 2.4.5 Noise-aware dynamic routing

For each clean image x and each noisy realization $x^{\left(n\right)}$, the routing network computes independent mixture weights,

$$\alpha (\overset{˜}{x})=\text{Router}(\overset{˜}{x}),\;\alpha ({\overset{˜}{x}}^{\left(n\right)})=\text{Router}({\overset{˜}{x}}^{\left(n\right)}),$$ (7)

which define realization-specific effective kernels,

$$W_{\mathrm{eff}}({\overset{˜}{x}}^{\left(n\right)})=\overset{K}{\underset{k=1}{\sum }}\alpha_{k}({\overset{˜}{x}}^{\left(n\right)})W_{k}.$$ (8)

Gradients from the noise-aware loss propagate through the shared routing network, implicitly discouraging kernel-selection strategies that are oversensitive to scanner-specific perturbations. Because both the actual and noise-corrupted realizations update the same routing parameters, the model learns kernel mixtures that are more stable across stochastic perturbations. Unlike conventional data augmentation, which perturbs inputs without explicitly influencing routing behavior, the proposed objective indirectly promotes robustness in kernel selection through shared optimization across multiple realizations of the same B-scan.

#### 2.4.6 Deployment efficiency

At inference time, noise sampling is disabled, and only a single clean forward pass is performed. Consequently, NA-DyCNN retains computational complexity and latency identical to those of the baseline dynamic CNN while exhibiting substantially improved robustness to scanner and cohort shift.

### 2.5 Training Protocol

All models were trained using the AdamW optimizer^26^ with weight decay $\lambda =10^{-4}$ and a batch size of 64. The learning rate schedule employed a linear warm-up from $10^{-6}$ to $1\times 10^{-3}$ over five epochs, followed by cosine annealing^27^ to $10^{-6}$ over the remaining training period. Class imbalance was addressed using inverse frequency weighting. Early stopping was applied based on balanced validation accuracy with a patience of 10 epochs and a maximum training budget of 100 epochs. For each architecture, the hyperparameters were selected based on the performance on the validation split of the source dataset and then fixed across all cross-dataset evaluations for that model. The held-out test sets and all external datasets were never used during model selection and were accessed only once for the final evaluation.

#### 2.5.1 Noise-aware hyperparameter sensitivity

The hyperparameters for the noise-aware dynamic CNN, speckle variability scale (σ), regularization weight (β), and number of Monte Carlo realizations (N), were tuned using only the source-dataset-balanced validation split, with no access to the target-domain data. For the $D_{2}$-trained models, a grid search was performed over

σ∈{0.05,0.10,0.15,0.20},β∈{0.10,0.30},N∈{1,2,4,6,8},

with all other architectural and optimization hyperparameters fixed. The model was selected based on the source domain validation accuracy.

**Fig. 4:**
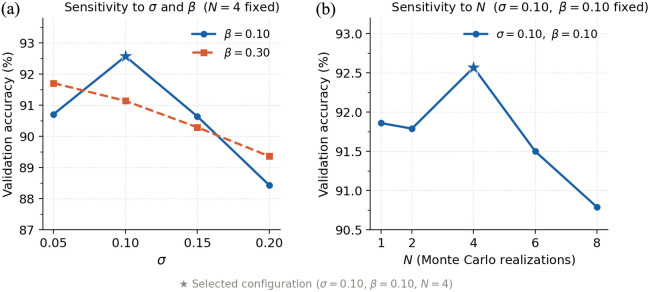
Hyperparameter sensitivity analysis on the OCT-C8 source-domain validation split. (a) Validation accuracy as a function of speckle scale σ for β∈{0.10,0.30} with N=4 fixed. (b) Validation accuracy as a function of the number of Monte Carlo realizations N with σ=0.10 and β=0.10 fixed. The selected configuration (σ=0.10, β=0.10, N=4) achieved the best validation performance among the explored configurations and was used for all cross-dataset evaluations.

Figure 4 shows the resulting sensitivity analysis for the $D_{2}$ source dataset. Moderate perturbation strengths yielded the strongest performance, with the validation accuracy peaking at σ=0.10 for β=0.10. Increasing σ beyond this point led to a consistent decline in performance, suggesting that excessively strong perturbations introduce unrealistic image corruption that degrades the diagnostically relevant retinal structure. Increasing the number of Monte Carlo realizations improved the performance up to N=4, after which the gains saturated and slightly declined despite the increased computational cost. An identical tuning protocol was applied to the $D_{1}$-trained models. The initial coarse search identified the optimal region near σ=0.05, and a subsequent finer local search yielded the final configuration σ=0.06 and β=0.12. For both training datasets, the validation performance was maximized at N=4. Consequently, the final hyperparameters were fixed to (σ=0.06,β=0.12,N=4) for $D_{1}$ and (σ=0.10,β=0.10,N=4) for $D_{2}$ for all subsequent cross-dataset evaluations.

When the target scanner is entirely unknown at training time, a conservative default configuration (σ=0.10, β=0.10, N=4) selected using source-domain validation data provides a practical operating point, as the sensitivity analysis in Fig. 4 demonstrates stable performance across moderate perturbation strengths. More broadly, the proposed framework is intended to improve robustness to acquisition-related variability arising from scanner hardware, image resolution, reconstruction algorithms, and vendor-specific preprocessing pipelines rather than to model a single scanner’s noise distribution exactly.

### 2.6 Evaluation Protocol

1. **Cross-dataset generalization (strict zero shot).** Models are trained independently on $D_{1}$ or $D_{2}$ and evaluated on all remaining datasets under strict patient-level splits. Fine-tuning, weight adaptation, or batch norm updating was not performed on any target domain. The only permitted target domain access is the 10% labeled subset used exclusively for Mondrian conformal calibration in the site-specific safety analysis. For $D_{1}/D_{2}\to D_{3}$ evaluation, the DME output logit was discarded because no corresponding labels exist in NEH. The remaining logits for CNV, Drusen, and normal were renormalized prior to computing performance metrics.
2. **Performance metrics.** We report performance metrics, such as accuracy, macro sensitivity, macro specificity, and recall-weighted $F_{2}$ score (β=2, weighted average) to prioritize pathological recall in screening.
3. **Reliability and uncertainty metrics.** Model reliability was assessed using Cohen’s κ^28^ and expected calibration error (ECE)^29^ computed with 15 equal-mass bins.
  The uncertainty quality was evaluated using the error–uncertainty metric (Err.Unc., %), which measures the fraction of misclassified samples lying above the 75th percentile of predictive entropy, indicating correct uncertainty attribution.
  Err.Unc.=∑i1(y^i≠yi∧H(pi)>Q75)∑i1(y^i≠yi)×100, (9)
  where $H(p_{i})$ denotes the predictive entropy of sample i and $Q_{75}$ is the 75th percentile of entropy over the test set. Higher values indicate that model errors are preferentially associated with high uncertainty, reflecting safer clinical behavior.
4. **Mondrian conformal prediction.** To obtain finite-sample uncertainty guarantees under dataset shift, we apply class-conditional (Mondrian) conformal prediction.^30^^,^^31^ For each class y, nonconformity scores are defined as
  $$\alpha_{i}^{y}=1-p_{i,y},$$ (10)
  computed on calibration samples whose true label is y. Class-specific thresholds $q_{y}$ are obtained from the (1−ϵ) quantile of $\left\{\alpha_{i}^{y}\right\}$, yielding prediction sets,
  $$C(p)=\{y:1-p_{y}\leq q_{y}\},$$ (11)
  This guarantees a marginal class-conditional coverage of at least 1−ϵ=95%.
  Two deployment scenarios were evaluated: (a) zero-shot, where source-domain validation data are used for calibration and applied directly to target domains; and (b) site-specific, where 10% of the target domain patients are used for calibration with enforced $\min(n_{y})\geq 5$ samples per class, reflecting realistic semisupervised clinical adaptation.
  We report the conformal coverage, average set size, and singleton rate as performance metrics.
5. **Statistical validation.** The observed improvements were validated using hierarchical bootstrap resampling^32^ with 2000 iterations, stratified at both the seed and patient levels. We report 95% confidence intervals for differences in accuracy, weighted $F_{2}$, and selective risk at 80% coverage (Risk@80%). Statistical significance was declared when the confidence interval excluded zero. All results are reported as mean ± standard deviation across three random seeds.

#### 2.6.1 Selective risk at fixed coverage

To evaluate performance under abstention, we compute selective risk at 80% coverage (Risk@80%).^33^ Predictions were ranked by confidence (low predictive entropy), and the top 80% were retained. Risk@80% is the classification error for this subset:

Risk@80%=1|S0.8|∑i∈S0.81(y^i≠yi),

where $S_{0.8}$ denotes the retained set. Lower values indicate safer, high-confidence predictions.

## 3 Results

We evaluated the clinical readiness of the proposed NA-DyCNN for cross-dataset deployment through four lenses: (1) diagnostic accuracy across dataset cohorts, establishing baseline competence; (2) formal safety via site-specific Mondrian conformal prediction; (3) reliability of uncertainty estimates under dataset shift; and (4) computational efficiency, enabling real-time deployment. This progression reflects deployment priorities; a model must diagnose accurately, fail safely, and operate within realistic clinical hardware constraints.

### 3.1 Cross-Dataset Diagnostic Competence

Table 3 reports the diagnostic generalization across four cross-dataset transfer scenarios: $D_{1}\to D_{2}$, $D_{1}\to D_{3}$, $D_{2}\to D_{1}$, and $D_{2}\to D_{3}$, capturing a spectrum of acquisition-protocol, population, and annotation variability across hospital cohorts. Metrics were reported in a clinically prioritized order: weighted $F_{2}$-score (recall-weighted), accuracy, sensitivity, and specificity.

**Table 3:** Cross-dataset diagnostic competence under domain shift (mean ± std over three seeds). Bold indicates the best lightweight model. All datasets used are summarized in Table 1.

Train → Test	Model	Weighted $F_{2}$ score (%)↑	Accuracy (%)↑	Sensitivity (%)↑	Specificity (%)↑
$D_{1}\to D_{3}$	Basic CNN	69.65±2.4	71.27±1.9	68.91±2.3	85.57±0.7
	Dynamic CNN	66.78±8.9	69.33±8.3	65.91±6.6	84.62±3.0
	**NA-DyCNN**	72.54±2.1	74.22±2.5	70.66±1.1	86.55±0.6
	MobileNetV3	71.88±3.2	72.84±3.7	70.08±1.5	85.29±1.0
	ResNet18 (ref)	76.35±3.5	77.75±3.2	73.57±2.9	87.41±1.3
$D_{2}\to D_{3}$	Basic CNN	62.15±5.5	62.90±5.2	64.54±4.5	82.15±1.9
	Dynamic CNN	71.06±4.0	71.80±4.4	72.13±1.7	85.90±1.8
	**NA-DyCNN**	74.90±2.3	75.49±2.3	74.52±1.2	87.34±0.6
	MobileNetV3	62.92±6.7	63.93±6.4	62.65±3.4	81.65±1.8
	ResNet18 (ref)	78.34±1.9	78.52±1.8	78.08±2.3	88.74±0.9
$D_{2}\to D_{1}$	Basic CNN	87.01±2.2	86.97±2.2	86.90±2.3	95.83±0.7
	Dynamic CNN	89.98±1.9	89.95±2.0	90.75±2.2	96.83±0.7
	**NA-DyCNN**	92.89±0.4	92.87±0.4	93.02±0.6	97.71±0.1
	MobileNetV3	90.75±1.3	90.72±1.3	90.60±1.2	97.09±0.4
	ResNet18 (ref)	94.41±0.9	94.40±0.9	94.61±0.9	98.21±0.3
$D_{1}\to D_{2}$	Basic CNN	91.18±0.3	91.21±0.3	91.21±0.3	97.07±0.1
	Dynamic CNN	92.05±0.6	92.09±0.6	92.09±0.6	97.36±0.2
	**NA-DyCNN**	92.29±0.6	92.33±0.6	92.33±0.6	97.44±0.2
	MobileNetV3	91.96±1.2	91.98±1.2	91.98±1.2	97.33±0.4
	ResNet18 (ref)	94.33±0.5	94.34±0.5	94.34±0.5	98.11±0.2

Across all four transfer settings, NA-DyCNN consistently achieved the highest recall-weighted $F_{2}$-scores among the lightweight models, indicating superior preservation of pathological sensitivity under dataset shift. Performance gains are most pronounced under $D_{1}\to D_{3}$ and $D_{2}\to D_{3}$, demonstrating that the proposed architecture maintains diagnostic competence even under substantial cross-site variability.

### 3.2 Safety under Cross-Site Domain Shift

Beyond diagnostic competence, we assessed clinical safety under cross-site domain shifts using Mondrian conformal prediction (MCP), a class-conditional uncertainty quantification framework that provides distribution-free coverage guarantees. We evaluated two deployment regimes: no-adaptation (zero-shot MCP using source-domain calibration only) and site-specific MCP with 10% labeled target-domain calibration, reflecting realistic clinical rollout conditions. The results are presented in Table 4.

**Table 4:** Mondrian conformal prediction safety under cross-site domain shift. Comparison of zero-shot (no target calibration) and site-specific (10% target calibration) models. Coverage is reported in %. Bold indicates the best lightweight model. All datasets used are summarized in Table 1.

Shift	Model	No adaptation (zero-shot)			Site-specific (10% calibration)		
		Coverage (%) ↑	Set size ↓	Singleton (%) ↑	Coverage (%) ↑	Set Size ↓	Singleton (%) ↑
$D_{1}\to D_{2}$	Basic CNN	91.9±0.2	1.06±0.04	93.4±2.9	95.0±0.7	1.12±0.01	88.0±0.7
	Dynamic CNN	92.1±0.4	1.02±0.01	95.0±0.8	95.0±1.1	1.08±0.06	91.4±4.7
	**NA-DyCNN**	91.9±0.4	1.00±0.01	96.8±0.8	95.2±0.8	1.08±0.04	91.7±4.0
	MobileNetV3	93.3±0.7	1.10±0.03	90.5±2.5	95.1±0.2	1.26±0.22	75.3±20.0
	ResNet18 (ref)	93.5±0.1	1.00±0.01	96.8±0.7	95.3±0.9	1.02±0.03	96.9±1.5
$D_{1}\to D_{3}$	Basic CNN	85.4±7.5	1.30±0.22	70.1±21.1	95.7±0.8	1.87±0.07	26.0±2.9
	Dynamic CNN	75.4±3.6	1.04±0.01	94.8±0.6	95.4±0.4	1.91±0.11	20.7±2.7
	**NA-DyCNN**	79.4±3.1	1.08±0.13	90.1±12.1	95.1±0.7	1.79±0.16	30.2±7.9
	MobileNetV3	88.1±4.1	1.53±0.09	49.5±11.2	95.4±0.7	2.20±0.29	18.0±1.1
	ResNet18 (ref)	79.7±4.1	1.04±0.02	95.4±2.6	94.7±0.4	1.74±0.03	29.5±3.8
$D_{2}\to D_{1}$	Basic CNN	96.52±0.32	1.37±0.12	67.14±9.32	95.62±0.82	1.33±0.10	70.14±7.77
	Dynamic CNN	96.07±0.32	1.18±0.07	82.99±6.41	95.64±0.63	1.18±0.11	83.05±9.33
	**NA-DyCNN**	96.45±0.18	1.10±0.02	89.94±1.80	95.48±0.68	1.08±0.03	91.60±2.48
	MobileNetV3	96.31±0.19	1.25±0.08	77.04±6.60	95.47±0.81	1.20±0.06	81.12±5.80
	ResNet18 (ref)	95.65±0.26	1.03±0.02	96.24±0.87	95.15±1.23	1.02±0.04	96.5±1.95
$D_{2}\to D_{3}$	Basic CNN	84.62±3.69	1.71±0.12	42.40±8.12	92.12±6.14	1.90±0.37	31.26±18.32
	Dynamic CNN	84.15±2.87	1.61±0.16	47.26±10.76	95.14±3.46	1.90±0.35	29.90±13.52
	**NA-DyCNN**	84.60±2.35	1.37±0.09	64.78±9.30	94.87±3.09	1.84±0.17	31.00±7.53
	MobileNetV3	83.59±5.85	1.66±0.12	47.04±10.33	92.93±5.13	2.18±0.34	16.05±13.86
	ResNet18 (ref)	83.89±4.04	1.18±0.16	82.19±15.80	94.61±2.89	1.74±0.21	39.32±11.24

**Why site-specific calibration is necessary:** Table 4 shows that the effect of cross-dataset evaluation on conformal prediction strongly depends on the severity of the dataset mismatch. Under the $D_{1}\to D_{2}$ transfer, all lightweight models maintained coverage relatively close to the nominal 95% target, even without target-domain calibration, indicating a relatively limited distribution mismatch. By contrast, the more challenging transfers involving $D_{3}$ exhibit substantial zero-shot under-coverage and inflated prediction sets, particularly for models without noise-aware training. For example, under the severe $D_{2}\to D_{3}$ shift, zero-shot deployment yielded only 84.6% coverage for NA-DyCNN, demonstrating the violation of exchangeability assumptions under a strong domain shift. However, calibrating the MCP using only 10% of the labeled target-domain patients restored coverage to ∼95% while maintaining compact prediction sets. This demonstrates that minimal site-specific calibration is sufficient to recover the formal safety guarantees. Across the severe transfer settings, NA-DyCNN consistently achieved smaller prediction sets and higher singleton rates than other lightweight baselines after calibration, indicating more decisive and high-confidence predictions under cross-site variability. Empirically, larger calibration fractions further improved coverage and reduced the set size but were not pursued to avoid increasing the clinical labeling burden, making the 10% setting a practical accuracy-cost trade-off for deployment.

To examine class-level reliability, Table 5 reports per-class Mondrian conformal prediction performance for the representative $D_{1}\to D_{2}$ transfer setting. Consistent with the aggregate results, NA-DyCNN generally produced the most compact prediction sets and among the highest singleton rates across disease categories while maintaining coverage close to the nominal target. Similar trends were observed for the remaining transfer settings and are provided in Tables S1–S3 in Supplementary Material.

**Table 5:** Per-class Mondrian conformal prediction performance for the $D_{1}\to D_{2}$ transfer scenario. Zero-shot corresponds to source-only calibration, whereas site-specific uses 10% target-domain calibration. Coverage and singleton values are reported in %. Datasets used are summarized in Table 1. Bold indicates the best lightweight model within each class.

Class	Model	No adaptation (zero-shot)			Site-specific (10% calibration)		
		Coverage (%) ↑	Set size ↓	Singleton (%) ↑	Coverage (%) ↑	Set size ↓	Singleton (%) ↑
CNV	Basic CNN	92.1±0.7	1.07±0.03	92.0±2.5	93.8±1.5	1.08±0.02	91.6±1.0
	Dynamic CNN	93.0±1.0	1.04±0.01	94.6±0.9	94.1±1.4	1.04±0.05	94.9±3.0
	**NA-DyCNN**	93.1±0.6	1.03±0.02	95.8±1.5	94.3±1.7	1.06±0.04	94.3±3.9
	MobileNetV3	94.9±1.1	1.08±0.05	92.0±5.3	95.6±1.4	1.57±0.53	47.9±47.4
	ResNet18 (ref)	94.5±1.2	1.02±0.01	97.4±0.2	94.7±2.6	1.01±0.03	97.3±0.7
DME	Basic CNN	96.9±0.1	1.02±0.01	97.9±1.1	95.3±1.1	1.10±0.01	89.7±1.4
	Dynamic CNN	97.0±0.1	1.00±0.00	98.1±0.3	96.0±0.6	1.05±0.03	94.1±2.4
	**NA-DyCNN**	96.9±0.3	1.00±0.00	98.7±0.3	96.6±0.3	1.07±0.03	92.6±2.5
	MobileNetV3	97.6±0.4	1.02±0.01	97.8±0.6	95.3±0.1	1.11±0.07	89.5±6.9
	ResNet18 (ref)	97.2±0.3	1.00±0.00	98.9±0.2	96.0±0.5	1.00±0.02	98.6±0.9
DRUSEN	Basic CNN	96.8±0.5	1.06±0.02	94.0±2.0	95.8±1.4	1.16±0.01	84.3±1.0
	Dynamic CNN	97.1±0.2	1.04±0.02	95.5±1.8	95.3±2.4	1.14±0.11	85.9±10.0
	**NA-DyCNN**	96.6±0.2	1.02±0.01	97.3±1.0	95.7±2.0	1.11±0.08	89.2±7.3
	MobileNetV3	96.9±0.8	1.07±0.03	93.4±3.1	94.9±1.4	1.23±0.16	78.8±13.4
	ResNet18 (ref)	97.4±0.4	1.02±0.01	97.5±0.5	95.9±0.7	1.05±0.03	95.1±3.1
NORMAL	Basic CNN	81.8±1.4	1.07±0.08	89.9±5.9	95.1±0.1	1.14±0.03	86.3±2.6
	Dynamic CNN	81.3±0.5	1.00±0.01	91.9±0.6	94.8±0.5	1.09±0.04	90.7±4.3
	**NA-DyCNN**	81.0±0.7	0.97±0.02	95.6±0.6	94.2±0.5	1.09±0.03	90.7±2.8
	MobileNetV3	83.7±1.1	1.22±0.03	78.9±2.9	94.5±1.0	1.15±0.14	85.1±13.3
	ResNet18 (ref)	84.8±1.2	0.96±0.03	93.4±2.2	94.8±1.8	1.02±0.03	96.5±1.7

#### 3.2.1 Uncertainty calibration quality

As shown in Table 6, the uncertainty calibration behavior of the NA-DyCNN is shift-dependent. Under the more severe transfer settings involving $D_{3}$, NA-DyCNN consistently achieved the highest Cohen’s κ among the lightweight models together with the strongest uncertainty attribution performance (Err.Unc), indicating that prediction errors were more consistently associated with elevated predictive uncertainty. Under the milder $D_{1}\to D_{2}$ and $D_{2}\to D_{1}$ shifts, the lightweight models achieved broadly comparable calibration quality, with MobileNetV3 obtaining the lowest ECE. These results suggest that the proposed noise-aware routing mechanism provides the largest reliability benefits under substantial cross-site variability rather than mild domain shifts. More generally, the results indicate that calibration quality alone does not fully capture deployment reliability under domain shift, thereby motivating the complementary use of conformal prediction to obtain formal uncertainty guarantees independent of probabilistic calibration quality.

**Table 6:** Uncertainty calibration quality under cross-site domain shift (mean ± std over three seeds). Bold text indicates the best performance among the lightweight models. Datasets used are summarized in Table 1.

Shift	Model	Cohen’s κ ↑	ECE ↓	Err. Unc (%) ↑
$D_{2}\to D_{1}$	Basic CNN	0.812 ± 0.030	0.095 ± 0.002	64.1 ± 3.1
	Dynamic CNN	0.855 ± 0.028	0.084 ± 0.006	69.5 ± 6.8
	**NA-DyCNN**	**0.896 ± 0.006**	0.083 ± 0.009	**79.8 ± 1.6** ^a^
	MobileNetV3	0.865 ± 0.018	**0.046 ± 0.004**	77.7 ± 2.6
	ResNet18 (Ref)	0.918 ± 0.013	0.058 ± 0.008	79.7 ± 3.3
$D_{2}\to D_{3}$	Basic CNN	0.436 ± 0.067	0.056 ± 0.038	37.6 ± 4.0
	Dynamic CNN	0.558 ± 0.056	**0.035 ± 0.007**	47.4 ± 5.2
	**NA-DyCNN**	**0.608 ± 0.029**	0.042 ± 0.002	**48.0 ± 1.4**
	MobileNetV3	0.434 ± 0.074	0.158 ± 0.048	36.3 ± 3.8
	ResNet18 (Ref)	0.655 ± 0.029	0.021 ± 0.005	51.3 ± 3.1
$D_{1}\to D_{2}$	Basic CNN	0.883 ± 0.004	0.117 ± 0.015	57.8 ± 4.7
	Dynamic CNN	0.895 ± 0.008	0.097 ± 0.004	**61.5 ± 2.4**
	**NA-DyCNN**	**0.898 ± 0.008**	0.098 ± 0.009	59.7 ± 5.3^a^
	MobileNetV3	0.893 ± 0.015	**0.091 ± 0.005**	49.0 ± 7.5
	ResNet18 (Ref)	0.925 ± 0.006	0.106 ± 0.009	57.7 ± 4.3
$D_{1}\to D_{3}$	Basic CNN	0.540 ± 0.028	0.063 ± 0.032	41.3 ± 1.8
	Dynamic CNN	0.512 ± 0.110	0.070 ± 0.036	39.6 ± 4.3
	**NA-DyCNN**	**0.580 ± 0.030**	0.050 ± 0.014	44.4 ± 2.7
	MobileNetV3	0.557 ± 0.046	**0.037 ± 0.005**	**45.3 ± 4.6**
	ResNet18 (Ref)	0.629 ± 0.047	0.084 ± 0.017	52.1 ± 6.5

**Cohen’s** κ, inter-rater reliability beyond chance; **ECE**, expected calibration error (15 bins); **Err.Unc** (%), fraction of misclassified samples exceeding the 75th percentile of predictive entropy, indicating correct uncertainty attribution.

a Indicates that the proposed approach outperforms ResNet18 on that metric

### 3.3 Qualitative Error Analysis

**Fig. 5:**
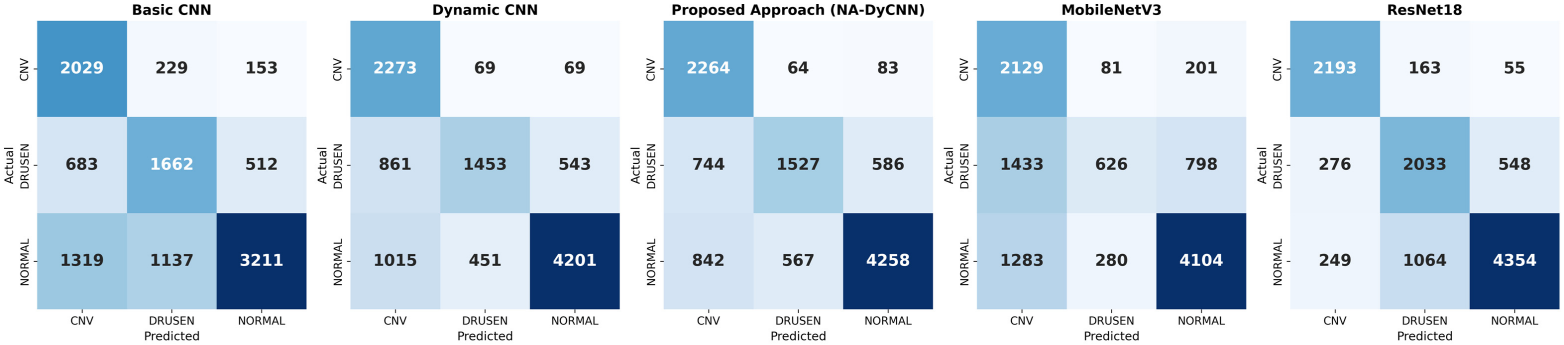
Confusion matrices for the cross-dataset shift ($D_{2}\to D_{3}$). For each architecture, the model instance corresponding to the median macro-F1 score across three independent seeds is visualized.

Figure 5 reveals that Drusen remains the dominant source of diagnostic ambiguity under severe cross-dataset shift ($D_{2}\to D_{3}$), consistent with its known morphological overlap with both early neovascular changes and healthy retinal appearance. All lightweight models exhibited bidirectional confusion between Drusen and CNV, as well as Drusen and normal, highlighting the intrinsic difficulty of this class under a scanner-induced domain shift.

Importantly, NA-DyCNN does not merely improve aggregate performance but reshapes the error structure in clinically meaningful ways. Compared with the standard dynamic CNN, NA-DyCNN reduces hazardous Drusen → CNV false positives from 861 to 744 cases (−13.6%) while simultaneously increasing Drusen recall (1453→1527 correct classifications), indicating improved sensitivity without sacrificing specificity. In parallel, NA-DyCNN also lowers Normal → CNV false alarms (1015→842), reducing unnecessary referrals in screening scenarios.

By contrast, MobileNetV3 exhibited a pronounced representation collapse under scanner shift, misclassifying more than half of Drusen scans as CNV and achieving only 21.9% Drusen sensitivity, showcasing the fragility of aggressive channel reduction strategies under the domain shift.

Although ResNet18 achieves the highest Drusen recall (2033 correct classifications), it does so at nearly 28× the parameter count of NA-DyCNN. Therefore, the proposed approach offers a favorable robustness–efficiency trade-off, substantially mitigating clinically hazardous confusions while retaining lightweight inference suitable for point-of-care OCT deployment.

### 3.4 Computational Efficiency for Clinical Deployment

**Fig. 6:**
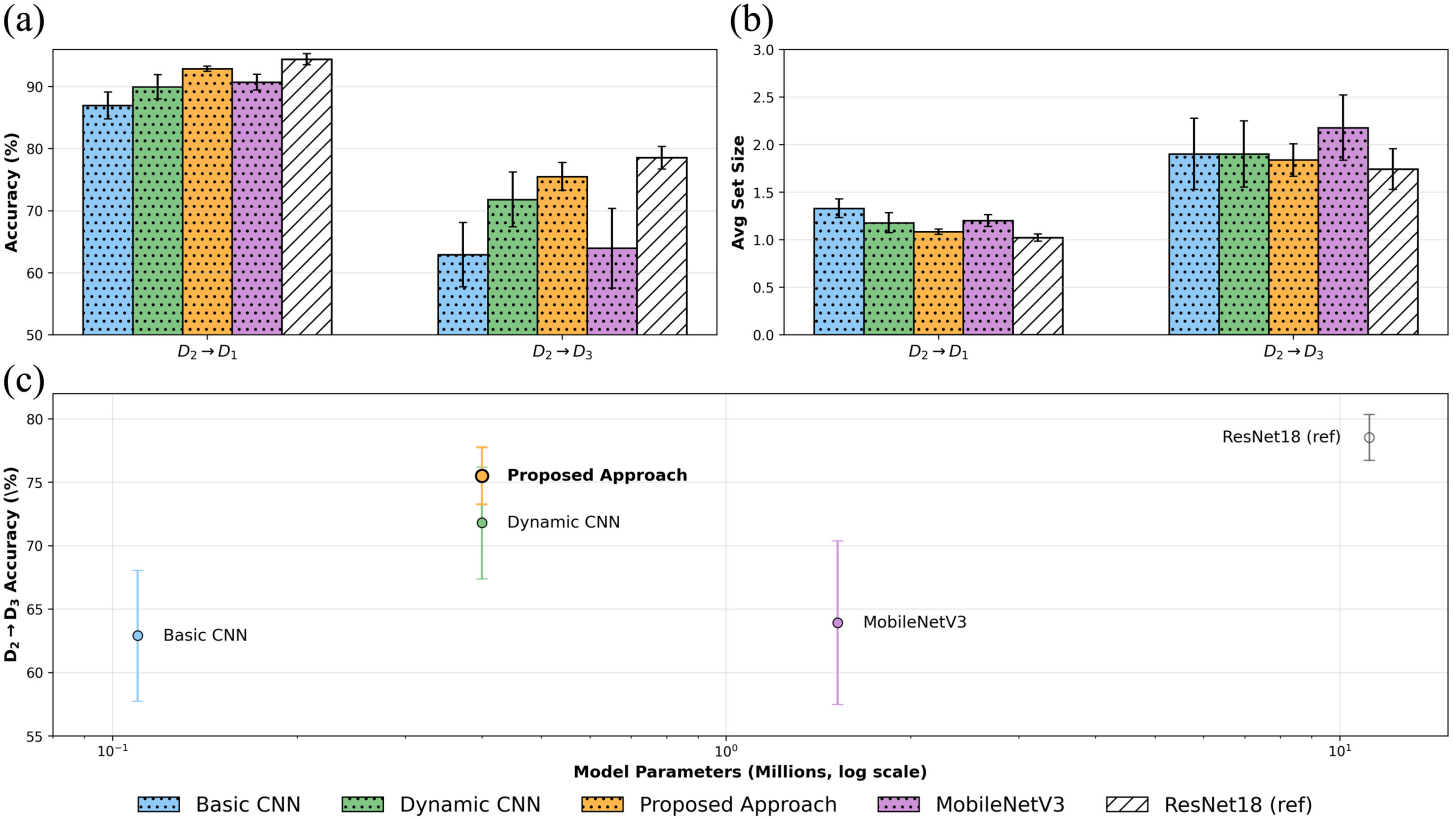
Clinical readiness metrics. (a) Diagnostic accuracy ($D_{2}\to D_{1}$, $D_{2}\to D_{3}$) with mean ± std over three seeds. (b) Average Mondrian conformal set size under site-specific 10% calibration (ϵ=0.05), reflecting clinical deployment realism. Lower set size = more decisive prediction. (c) Accuracy-efficiency Pareto frontier: NA-DyCNN achieves an optimal balance among lightweight models. ResNet18 (hollow markers) shown as high-capacity reference only.

Figure 6(c) shows that NA-DyCNN lies on the accuracy–efficiency Pareto frontier among lightweight models, achieving 75.5% accuracy on the most severe cross-site shift ($D_{2}\to D_{3}$) with only 0.40 M parameters and 0.53 ms inference latency on an NVIDIA GB 10 GPU. This corresponds to a 27.9× parameter reduction compared with ResNet18 (11.2M parameters) while retaining 95.6% of its diagnostic accuracy.

These results indicate that NA-DyCNN offers a favorable trade-off between predictive performance and computational cost, making it suitable for deployment in resource-constrained clinical environments, such as portable OCT workstations or edge-based screening systems, where memory footprint and latency are critical constraints.

### 3.5 Statistical Validation of Performance Gains

We assess robustness using hierarchical bootstrap resampling (2000 iterations, patient-stratified) across random seeds and the test subjects. We report differences in selective risk at 80% coverage (Risk@80%), recall-weighted $F_{2}$ score, and accuracy between NA-DyCNN and each baseline. Confidence intervals excluding zero indicate statistical significance.

Table 7 shows that the statistical significance of the NA-DyCNN improvements depends on the severity of the shift. For the mild $D_{1}\to D_{2}$ transfer, improvements over basic CNN, dynamic CNN, and MobileNetV3 are small, and the corresponding confidence intervals overlap with zero, indicating that the observed differences are not statistically significant. By contrast, for the more challenging $D_{2}\to D_{1}$ and $D_{2}\to D_{3}$ transfers, NA-DyCNN consistently achieves statistically significant reductions in selective diagnostic risk while simultaneously improving the recall-weighted $F_{2}$ score and classification accuracy.

**Table 7:** Hierarchical bootstrap significance under scanner shift. The primary metric is the selective risk at 80% coverage (Risk@80%). A negative Δrisk indicates safer, high-confidence predictions. All datasets used are summarized in Table 1.

Shift	Versus baseline	ΔRisk@80%	$\Delta F_{2}$	ΔAccuracy
$D_{1}\to D_{2}$	Basic CNN	−0.75[−2.13, 0.33]	+1.10[−0.12,1.92]	+1.11[−0.10,1.93]
	Dynamic CNN	+0.06[−1.28,1.03]	+0.24[−0.10,0.60]	+0.23[−0.10,0.58]
	MobileNetV3	−1.42[−2.74,0.79]	+0.33[−2.07,2.03]	+0.35[−2.02,2.02]
$D_{2}\to D_{1}$	Basic CNN	**−4.21**[−6.28,−2.19]	**+6.48**[4.00,9.19]	**+5.90**[3.17,8.46]
	Dynamic CNN	**−1.86**[−3.45,−0.26]	**+2.70**[0.11,5.51]	**+2.91**[0.82,5.22]
	MobileNetV3	**−1.72**[−2.76,−0.30]	**+2.56**[0.47,4.23]	**+2.14**[0.06,3.80]
$D_{2}\to D_{3}$	Basic CNN	**−13.54**[−25.69,−3.39]	**+11.48**[4.48,21.08]	**+12.74**[5.20,22.10]
	Dynamic CNN	**−3.50**[−4.96,−2.42]	**+2.99**[1.56,4.10]	**+3.70**[1.08,8.02]
	MobileNetV3	**−11.95**[−18.52,−6.57]	**+12.49**[7.92,15.80]	**+11.41**[6.38,16.64]

The largest gains are observed under the $D_{2}\to D_{3}$ transfer, where NA-DyCNN reduces the high-confidence error by 13.5 percentage points relative to the basic CNN, 11.95 percentage points relative to MobileNetV3, and 3.5 percentage points relative to dynamic CNN. These results suggest that the benefits of the proposed noise-aware objective become most pronounced under larger shifts, where robustness and calibration are particularly important for reliable deployment.

### 3.6 Mechanisms of Robustness: Ablation Study

To identify the individual contributions of each architectural and training component, we conducted an ablation study on the most challenging transfer ($D_{2}\to D_{3}$). Table 8 reports the performance (mean ± std over three seeds) as successive components are added, using site-specific Mondrian calibration with 10% target-domain patients. Variant (3) is included only as an intermediate ablation configuration to isolate the contribution of postacquisition speckle augmentation independently from the expectation-based noise-aware objective.

**Table 8:** Ablation study on severe cross-site shift ($D_{2}\to D_{3}$). Each component was added cumulatively to quantify its contribution to robustness. Descriptions of the datasets are summarized in Table 1.

Variant	DynConv	Post-Acq. Noise Aug	Noise-aware	Weighted $F_{2}$ (%) ↑	Accuracy (%) ↑	Cohen’s κ ↑	Set size ↓
(1) Basic CNN	×	×	×	62.15 ± 5.5	62.90 ± 5.2	0.434 ± 0.067	1.9 ± 0.37
(2) Dynamic CNN	✓	×	×	71.06 ± 4.0	71.80 ± 4.4	0.558 ± 0.056	1.89 ± 0.35
(3) Dynamic CNN with Noise Aug	✓	✓	×	72.85 ± 1.6	72.85 ± 1.8	0.572 ± 0.025	1.95 ± 0.11
(4) **Full NA-DyCNN**	✓	✓	✓	**74.90 ± 2.3**	**75.49 ± 2.3**	**0.608 ± 0.029**	**1.84 ± 0.17**

Values are mean ± std over three random seeds under the $D_{2}\to D_{3}$ shift with site-specific Mondrian conformal calibration (10% target-domain patients). DynConv, dynamic convolution kernels (K=4, attention routing); Post-Acq. Noise Aug., augmentation modeling residual scanner-export noise and texture variability in postprocessed OCT images; Noise-aware, expectation-based loss [Eq. (6)] with N=4 realizations.

Table 8 shows that dynamic convolution yields the largest absolute improvement, increasing weighted $F_{2}$ by ∼+8.91 percentage points and accuracy by +8.9 percentage points over the basic CNN baseline, together with a substantial increase in Cohen’s κ (from 0.43 to 0.56). Adding speckle-specific augmentation provides an additional boost of +1.8 percentage points in weighted $F_{2}$ and modest gains in accuracy and κ, although with a slightly higher set size. The full noise-aware objective produces the strongest overall gains, with a further +2.1 percentage point improvement in weighted $F_{2}$, +2.6 percentage points in accuracy, a+0.036 increase in κ, and the lowest set size (1.84), indicating more decisive and reliable high-confidence predictions under the cross-site domain shift.

These results indicate that robustness emerges from the combined effect of dynamic kernel adaptation, OCT-specific speckle augmentation, and expectation-based optimization over speckle perturbations rather than from any single component in isolation.

### 3.7 Analysis of Dataset Information in Routing Coefficients

To directly evaluate whether the proposed noise-aware objective reduces the dataset-specific information encoded in the routing decisions, we analyzed the routing coefficients (α) from the final dynamic convolution block for the dynamic CNN and NA-DyCNN. The $D_{1}$-trained model was utilized for this analysis because both $D_{1}$ and $D_{3}$ were acquired using Heidelberg Spectralis scanners; therefore, distinguishing between these two datasets would provide limited insight into scanner-related variability. Instead, the routing coefficients from $D_{2}$ and $D_{3}$ were evaluated, where $D_{2}$ (OCT-C8) contained images acquired from multiple scanner vendors and $D_{3}$ consisted of images acquired using a Heidelberg Spectralis scanner. Consequently, distinguishing $D_{2}$ from $D_{3}$ provides a more meaningful assessment of whether the routing coefficients retain dataset- and scanner-related information. A logistic regression classifier was trained to predict dataset-of-origin ($D_{2}$ versus $D_{3}$) using only the routing coefficients α. Five-fold stratified cross-validation was performed, and the results were averaged across three independent training seeds. Dynamic CNN achieved a mean dataset classification accuracy of 0.756±0.007, whereas NA-DyCNN achieved 0.733±0.008. Because a lower classification accuracy indicates that the dataset-of-origin is more difficult to infer from the routing coefficients, these results suggest that the proposed noise-aware objective reduces the dataset-specific information encoded within the routing space. This observation is consistent with the hypothesis that NA-DyCNN promotes more dataset-invariant routing behavior, which contributes to the improved cross-dataset generalization results reported in Table 8.

## 4 Discussion

This study addresses a key barrier to the deployment of deep learning for retinal OCT screening: the limited generalization of lightweight models across heterogeneous scanners and clinical sites. By incorporating postacquisition residual speckle variability into a dynamic convolution framework, NA-DyCNN improves cross-dataset robustness without increasing the inference cost or requiring any form of target-domain fine-tuning. From an imaging perspective, this approach explicitly accounts for the variability of OCT speckle statistics across acquisition systems. Speckle patterns arise from coherent interference of backscattered light and are influenced by optical system parameters and tissue microstructure; therefore, variations in scanner design and reconstruction pipelines naturally lead to differences in observed speckle distributions.^3^ However, scanner variability is not the sole source of distribution shift in real-world OCT deployment. Differences in patient demographics, acquisition protocols, export pipelines, and dataset curation strategies can substantially alter the target distribution. This is particularly evident in the $D_{1}\to D_{3}$ transfer setting, where both datasets use Heidelberg Spectralis devices but still exhibit a substantial cross-site distribution shift.

Across all external validation scenarios, NA-DyCNN achieved the best performance among lightweight models under strict zero-shot transfer, where no model parameters or normalization statistics were adapted to the target domain. Statistically significant gains are observed under the most severe shifts ($D_{2}\to D_{3}$ and $D_{2}\to D_{1}$). Ablation analysis confirmed that dynamic convolution provided the dominant performance improvement, whereas the expectation-based noise-aware objective further stabilized kernel routing against scanner-export speckle variability. These findings suggest that explicitly modeling speckle perturbations during training helps prevent the model from relying on scanner-specific texture signatures that do not generalize across acquisition systems.

Beyond diagnostic accuracy, NA-DyCNN improves clinical reliability, yielding higher Cohen’s κ and more compact conformal prediction sets after minimal site-specific calibration. Although the expected calibration error remains elevated under severe dataset shifts, this limitation is mitigated by the use of conformal prediction, which provides distribution-free safety guarantees independent of the probability calibration quality.

The under-coverage observed in the NEH cohort highlights the limitations of generalizability induced by the combined scanner, population, and protocol shift, highlighting the inherent difficulty of uncertainty quantification in real-world deployment. These findings are consistent with prior observations that OCT speckle patterns encode acquisition-dependent information related to both imaging physics and tissue scattering properties.^3^ As a result, models trained on a single dataset may inadvertently learn scanner-specific speckle statistics rather than pathology-relevant structures.

Recent lightweight architectures, including modern mobile CNNs (e.g., MobileNetV4), lightweight transformer models, and hybrid CNN-transformer architectures such as MobileViT, EfficientViT, and FastViT, have demonstrated strong performance across various computer vision tasks. However, the primary contribution of this study lies in the proposed noise-aware dynamic routing framework rather than the backbone architecture itself. A deliberate design choice in this study was to train all models from scratch rather than relying on large-scale pretrained models. Although transfer learning often improves performance, pretrained representations are typically learned from natural image datasets and may introduce biases that are not directly relevant to retinal OCT imaging. Moreover, transfer learning remains susceptible to domain shifts, making it difficult to disentangle the effects of pretraining from those of the proposed robustness framework. The proposed approach is architecture-agnostic and can be viewed as a general strategy for encouraging scanner-invariant feature utilization via noise-aware routing. Although this study focuses on modeling dataset-dependent speckle variability, other acquisition-related factors, such as resolution differences, reconstruction pipelines, contrast variations, and device-specific artifacts, may also contribute to domain shift. Future work could therefore investigate alternative augmentation and perturbation strategies within the same framework, as well as integration with modern lightweight CNN, transformers, and hybrid architectures.

Future work will also include validation on raw 12/16-bit OCT data to better disentangle physical speckle formation from vendor postprocessing artifacts, development of scanner-aware calibration strategies, and extension to volumetric OCT and additional vendor platforms. More broadly, the results support the emerging perspective that speckle should not be treated purely as a nuisance artifact but as an imaging characteristic whose statistical variability must be considered when developing robust OCT analysis algorithms.

Despite these limitations, NA-DyCNN retains an identical inference cost to standard dynamic CNNs while being nearly 28× smaller than ResNet18, making it well-suited for real-time deployment in portable and resource-constrained OCT screening environments.

## 5 Conclusion

We presented NA-DyCNN, a noise-aware dynamic convolutional framework for retinal OCT classification that integrates scanner-export residual speckle variability into the training objective within the clinical image-processing domain. By optimizing over multiple noise-corrupted realizations that approximate variations in OCT speckle statistics across acquisition systems, the model improves robustness to dataset shifts while preserving lightweight inference efficiency.

A comprehensive evaluation across three heterogeneous OCT cohorts demonstrated that NA-DyCNN consistently outperformed existing lightweight baselines and approached the performance of deeper residual networks at a fraction of the computational cost. Beyond accuracy, the proposed framework improves diagnostic reliability and conformal prediction efficiency under dataset shifts, supporting safer clinical deployment.

These findings suggest that explicitly accounting for speckle variability can improve the generalizability of deep learning models across scanners and clinical sites. More broadly, incorporating clinically observable imaging variability into adaptive convolutional architectures provides a promising pathway toward robust and deployable OCT screening systems in real-world healthcare environments.

## Supplementary Material

10.1117/1.JBO.31.8.086006.s01

## Data Availability

All datasets used in this study are publicly available from their respective sources. The UCSD retinal OCT dataset is available at: https://data.mendeley.com/datasets/rscbjbr9sj/2. The NEH retinal OCT dataset can be accessed at: https://data.mendeley.com/datasets/8kt969dhx6/2. The OCT-C8 dataset is available via Kaggle at: https://www.kaggle.com/datasets/obulisainaren/retinal-oct-c8. All datasets were used in accordance with their respective usage policies and licenses.
