# Supplementary material for: Noise-aware dynamic convolution for improved generalizability of retinal disease diagnosis using optical coherence tomography images
Source: J Biomed Opt. 2026 Aug 12;31(8):086006. doi: 10.1117/1.JBO.31.8.086006 (PMC13464582; doi:10.1117/1.JBO.31.8.086006)
Supplement: Supplementary file 1 [file JBO_031_086006_SD001.pdf]

Manuscript Number: JBO-260098P-1

Noise-Aware Dynamic Convolution for Improved Generalizability of Retinal Disease Diagnosis using Optical Coherence Tomography Images

Deeksha Chutani and Phaneendra K. Yalavarthy

1 Class-Conditional Mondrian Conformal Prediction Analysis

To complement the aggregate Mondrian conformal prediction (MCP) results reported in the main manuscript, this section provides a detailed class-level analysis for the remaining cross-dataset transfer settings. For each transfer scenario, we report class-wise coverage, average prediction set size, and singleton rate under both zero-shot deployment and site-specific calibration using 10% labeled target-domain data. These results provide additional insight into how uncertainty estimates behave across individual retinal disease categories under dataset shift.

Table S1. Per-class Mondrian conformal prediction performance for the $D_1 \rightarrow D_3$ transfer scenario. Zero-shot corresponds to source-only calibration, whereas site-specific uses 10% target-domain calibration. Coverage and singleton values are reported in %. Bold indicates the best lightweight model within each class.

**Class**	**Model**	**No Adaptation (Zero-Shot)**			**Site-Specific (10% Calibration)**		
		**Coverage (%) ↑**	**Set Size ↓**	**Singleton (%) ↑**	**Coverage (%) ↑**	**Set Size ↓**	**Singleton (%) ↑**
CNV	Basic CNN	**92.3±2.0**	1.08±0.05	92.3±4.9	96.8±0.6	**1.29±0.11**	**75.7±9.4**
	Dynamic CNN	92.0±1.9	1.03±0.03	96.4±2.5	**97.4±0.7**	1.36±0.06	67.6±6.5
	**NA-DyCNN**	91.6±2.1	**1.02±0.03**	**97.3±2.1**	96.9±0.4	1.31±0.03	72.4±2.5
	MobileNetV3	85.6±2.3	1.19±0.03	82.2±3.2	96.5±1.7	1.55±0.07	55.1±10.6
	ResNet18 (ref)	87.8±6.2	1.02±0.02	96.9±1.8	95.2±0.8	1.24±0.04	78.4±4.2
DRUSEN	Basic CNN	60.2±28.1	1.38±0.21	62.4±20.0	95.1±0.9	1.95±0.09	17.4±4.4
	Dynamic CNN	19.9±10.2	**1.06±0.04**	**90.2±3.2**	95.6±1.5	1.99±0.12	12.1±5.1
	**NA-DyCNN**	39.3±20.6	1.11±0.18	84.2±14.5	94.9±1.2	**1.88±0.12**	**19.8±6.2**
	MobileNetV3	**82.3±7.0**	1.54±0.09	48.1±10.0	**95.7±0.5**	2.14±0.27	16.3±4.5
	ResNet18 (ref)	41.0±14.7	1.08±0.05	90.0±5.8	94.1±1.4	1.75±0.01	28.8±3.8
NORMAL	Basic CNN	95.1±1.2	1.36±0.29	64.6±28.6	**95.5±1.2**	2.07±0.05	9.2±3.0
	Dynamic CNN	**96.3±2.9**	**1.03±0.01**	**96.5±1.1**	94.5±0.7	2.11±0.18	5.1±6.1
	**NA-DyCNN**	94.5±5.0	1.10±0.16	90.1±15.3	94.5±0.7	**1.94±0.25**	**17.4±13.7**
	MobileNetV3	92.0±5.9	1.67±0.14	36.3±16.5	94.8±1.4	2.51±0.43	3.0±2.1
	ResNet18 (ref)	95.8±5.2	1.02±0.02	97.5±2.0	94.8±0.4	1.95±0.05	9.1±6.3

Table S2. Per-class Mondrian conformal prediction performance for the $D_2 \rightarrow D_1$ transfer scenario. Zero-shot corresponds to source-only calibration, whereas site-specific uses 10% target-domain calibration. Coverage and singleton values are reported in %. Bold indicates the best lightweight model within each class.

Class	Model	No Adaptation (Zero-Shot)			Site-Specific (10% Calibration)		
		Coverage (%) ↑	Set Size ↓	Singleton (%) ↑	Coverage (%) ↑	Set Size ↓	Singleton (%) ↑
CNV	Basic CNN	96.9±0.6	1.35±0.09	68.3±7.1	95.1±0.3	1.27±0.08	73.9±6.6
	Dynamic CNN	96.5±0.3	1.20±0.02	80.1±1.8	95.1±0.1	1.16±0.07	84.2±6.3
	**NA-DyCNN**	**97.1±0.4**	**1.12±0.02**	**87.8±1.5**	**95.4±0.2**	**1.10±0.03**	**90.4±3.0**
	MobileNetV3	96.6±0.5	1.26±0.07	75.7±6.4	95.3±0.4	1.17±0.04	83.6±3.5
	ResNet18 (ref)	96.2±0.9	1.04±0.02	95.6±1.5	94.8±0.3	1.01±0.03	97.8±2.2
DME	Basic CNN	95.2±0.2	1.29±0.07	75.2±4.9	94.7±0.7	1.19±0.05	82.4±3.4
	Dynamic CNN	94.7±1.4	1.14±0.12	86.4±11.4	94.4±0.1	1.11±0.12	89.2±10.6
	**NA-DyCNN**	94.8±1.0	**1.07±0.02**	**93.4±1.5**	**95.0±0.2**	**1.04±0.02**	**96.0±1.4**
	MobileNetV3	**96.7±0.7**	1.15±0.05	87.3±4.1	94.6±0.5	1.09±0.03	92.2±2.5
	ResNet18 (ref)	95.3±0.2	1.02±0.03	96.7±1.8	94.8±0.5	1.00±0.03	97.5±0.8
DRUSEN	Basic CNN	96.5±0.6	1.45±0.20	59.6±15.6	94.8±0.8	1.32±0.15	69.8±12.4
	Dynamic CNN	96.0±1.3	1.20±0.09	80.7±8.5	95.0±0.4	1.14±0.10	85.9±9.1
	**NA-DyCNN**	**96.1±0.2**	**1.16±0.05**	**84.4±4.6**	**95.3±1.4**	**1.09±0.02**	**90.7±2.3**
	MobileNetV3	96.6±0.3	1.24±0.05	77.2±4.5	94.5±1.5	1.16±0.04	84.5±4.1
	ResNet18 (ref)	96.2±0.2	1.04±0.01	96.0±0.8	94.5±1.2	1.00±0.02	98.2±1.2
NORMAL	Basic CNN	**96.9±0.1**	1.43±0.19	62.9±14.3	94.7±0.4	1.29±0.15	73.2±13.1
	Dynamic CNN	96.3±0.6	1.13±0.13	87.7±11.4	95.2±0.8	1.11±0.17	88.7±14.4
	**NA-DyCNN**	96.5±0.3	**1.05±0.02**	**94.9±1.4**	**95.4±0.3**	**1.03±0.02**	**96.7±1.7**
	MobileNetV3	95.3±0.3	1.31±0.12	72.0±10.3	95.0±0.2	1.14±0.06	86.5±5.3
	ResNet18 (ref)	94.4±1.1	1.01±0.04	97.2±2.3	95.3±0.2	0.99±0.02	97.7±0.4

Table S3. Per-class Mondrian conformal prediction performance for the $D_2 \rightarrow D_3$ transfer scenario. Zero-shot corresponds to source-only calibration, whereas site-specific uses 10% target-domain calibration. Coverage and singleton values are reported in %. Bold indicates the best lightweight model within each class.

Class	Model	No Adaptation (Zero-Shot)			Site-Specific (10% Calibration)		
		Coverage (%) ↑	Set Size ↓	Singleton (%) ↑	Coverage (%) ↑	Set Size ↓	Singleton (%) ↑
CNV	Basic CNN	95.7±1.6	1.30±0.01	73.9±1.6	96.9±1.1	1.64±0.15	**52.9±8.3**
	Dynamic CNN	**97.9±2.6**	1.22±0.16	79.1±14.6	**97.0±1.7**	1.57±0.18	51.8±8.9
	**NA-DyCNN**	96.5±3.2	**1.11±0.01**	**89.5±1.1**	96.5±1.3	**1.55±0.12**	51.2±12.6
	MobileNetV3	89.9±9.7	1.38±0.19	70.2±14.9	95.8±1.6	1.90±0.15	35.7±14.0
	ResNet18 (ref)	93.1±2.7	1.05±0.04	94.7±4.1	96.4±0.8	1.24±0.06	80.8±4.6
DRUSEN	Basic CNN	**69.3±22.1**	1.79±0.05	33.5±5.0	95.5±0.7	2.21±0.15	12.4±3.6
	Dynamic CNN	66.2±22.1	1.74±0.04	34.6±4.0	95.1±2.1	1.93±0.32	**26.8±15.6**
	**NA-DyCNN**	56.6±12.2	**1.49±0.06**	**53.6±5.5**	**96.3±1.3**	**1.92±0.26**	25.6±16.7
	MobileNetV3	63.5±21.7	1.77±0.10	37.4±8.3	95.0±0.3	2.45±0.07	4.4±0.7
	ResNet18 (ref)	68.9±9.6	1.21±0.14	79.4±13.5	95.4±1.1	1.71±0.19	37.7±14.8
NORMAL	Basic CNN	87.6±8.4	1.83±0.21	33.5±12.7	95.0±1.2	2.44±0.17	9.4±4.8
	Dynamic CNN	87.4±15.1	1.70±0.24	40.1±14.8	**95.6±0.3**	2.13±0.44	**20.6±16.6**
	**NA-DyCNN**	**93.6±3.9**	**1.42±0.15**	**59.9±15.2**	95.1±0.2	**2.20±0.20**	12.5±8.5
	MobileNetV3	91.0±5.7	1.73±0.16	42.0±11.7	95.5±0.9	2.53±0.11	5.0±1.6
	ResNet18 (ref)	87.5±5.8	1.22±0.22	78.3±21.9	94.7±0.7	1.96±0.09	18.3±4.8

Across all transfer settings, the class-wise results are consistent with the aggregate findings reported in the main manuscript. For the relatively mild shifts (e.g., $D_1 \rightarrow D_2$ and $D_2 \rightarrow D_1$), site-specific calibration maintains coverage close to the nominal target while preserving compact prediction sets and high singleton rates. In contrast, the more challenging transfers involving D_3 exhibit substantial zero-shot under-coverage. After calibration using only 10% labeled target data,

coverage is restored to approximately 95%, but this is often accompanied by larger prediction sets and reduced singleton rates. This trade-off reflects the increased uncertainty required to recover valid conformal guarantees under severe domain shift. Across all settings, NA-DyCNN generally maintains smaller prediction sets and higher singleton rates than the other lightweight baselines while achieving comparable coverage, indicating more efficient uncertainty estimates under cross-domain deployment.
